# Acknowledgement of Reviewers

**DOI:** 10.1111/jdi.70176

**Published:** 2025-12-05

The publication of invaluable papers in the *Journal of Diabetes Investigation* depends on the prompt, careful review of submitted manuscripts. We would like to thank the Editorial Board members, the International Editorial Board members, the Assistant Editorial Board members, and the following experts for reviewing manuscripts from September 1, 2024 to August 31, 2025.

A. Seval Ozgu‐Erdinc

Abazar Roustazadeh

Abdullah Tuncay Demiryurek

Abolfazl Bahrami

Adeleh Khodabakhshi

Ahmed N. Kaftan

Ai Harashima

Aiko Arimura

Akihiko Narisada

Akihiro Hamasaki

Akihiro Isogawa

Akinobu Nakamura

Akinori Hayashi

Akio Kuroda

Akira Ishii

Akira Nishiyama

Akira Okada

Akira Shimada

Alexander Berezin

Alpesh Goyal

Altaisaikhan Khasag

Ami Fukunaga

Amin Mansoori

Amirmohammad Khalaji

Andong He

Andrew Dwyer

Ansu Basu

Apiradee Sriwijitkamol

Arnon Wiznitzer

Asaad Ma Babker

Atsuhito Saiki

Atsuhito Tone

Atsushi Goto

Atsushi Obata

Baohui Xu

Bei Xu

Bettina Gohlke

Bo Zhang

Bradley J. Macintosh

Brian Dewar

Carrie Breton

Catharina M. C. Mels

Catherine Knight Agarwal

Cc Kuo

C.‐H. Lai

Chaicharn Deerochanawong

Chao Xu

Chaofan Geng

Chaofan Wang

Cheli Melzer Cohen

Cheng Hu

Chia‐Huei Lin

Chia‐Hung Lin

Chia‐Luen Huang

Chieh‐Hua Lu

Chieko Murakami

Chieko Suzuki

Chih‐Yuan Wang

Chi‐Ming Chan

Chin‐Hsiao Tseng

Chloe Cheung

Chong Hwa Kim

Chung‐Ze Wu

Chun‐Heng Kuo

Daiji Kawanami

Daisuke Chujo

Daisuke Miyamori

Daisuke Sugiyama

Daisuke Tanaka

Daisuke Tsuriya

Damu Tang

Dan Kawamori

Daniela Di Lisi

Danying Deng

Das Sasmita

David A. Sacks

David Lui

Deyan Chen

Amir Aliramezani

Arian Rahimi

Zhang Saisai

E. Bonora

Eiji Kawasaki

Ekaterini Chatzaki

Elizabeth F. Sutton

Elly Lilianty Sjattar

Emi Ushigome

Emiri Miura‐Yura

Enrique Gea‐Izquierdo

Enrique Reyes‐Munoz

Eri Ikeguchi

Eri Toda Kato

Erik Melen

Eun Jung Choi

Eun Young Lee

Fadong Li

Feng Chih Kuo

Fengzhu Zhou

Frederic Domenge

Fumihiko Takeuchi

Gang Yuan

Garyfallia Pepera

Giuseppe Penno

Giuseppe Pugliese

Guang‐Da Xiang

Guanhu Yang

Gulali Aktas

Guobin Su

Habibollah Esmaeily

Hai Fang

Hande Atalay

Haosen Zhao

Haowei Liang

Haoyong Yu

Harn‐Shen Chen

Haruki Momma

Haruya Ohno

Hayato Tanabe

Hechen Shen

Hideaki Kaneto

Hideki Hayashi

Hidenori Fukuoka

Hidenori Koyama

Hideto Yonekura

Hideyoshi Kaga

Hiraku Kameda

Hiroaki Okazaki

Hiroaki Satoh

Hiroaki Yagyu

Hirokazu Takahashi

Hiroki Mizukami

Hiroki Teragawa

Hiroko Takaike

Hiromichi Wakui

Hiroshi Nomoto

Hiroshi Okada

Hiroshi Sakaue

Hiroto Furuta

Hiroyuki Nodera

Hiroyuki Umegaki

Hisafumi Yasuda

Hisashi Adachi

Hisashi Yokomizo

Hitomi Nakayama

Hitoshi Nishizawa

Honglei Hu

Hongsong Zhang

Ht Wu

Huabin Wang

Huiyun Wang

Ichiro Horie

Ichiro Nojima

Ichiro Yamauchi

Ievgeniia Burlaka

Ikuyo Ichi

Il Rae Park

Isa Sincer

I‐Te Lee

Jack Ng

Jack Yanovski

Jae Hyeon Kim

Jae‐Han Jeon

James J. Yahaya

Jer‐Ming Chang

Jia Liang Kwek

Jialin Han

Jia‐Long Chen

Jian Zhou

Jianming Wu

Jiaqing Shao

Jiemin Pan

Jiesi Wang

Jiming Fang

Jin Zhou

Jinbiao Zhang

Jing Xu

Jocelyn J. Drinkwater

Joonyub Lee

Joshua Miller

Julie A. Wagner

Jun Eguchi

Jun Shirakawa

Junichiro Irie

Junji Fujikura

Junji Kozawa

Junji Shinoda

Junko Sato

Junnosuke Miura

Kaijian Hou

Kana Miyake

Kanako Ohkuma

Kanitta Chamroonsawasdi

Kaori Ikeda

Karin Rådholm

Katarzyna Cyganek

Katsuhiko Hagi

Katsuhito Mori

Katsutaro Morino

Kaya Ishizawa

Kayo Waki

Kazuhiko Sakaguchi

Kazuhiro Omori

Kazuhiro Sugimoto

Kazuma Takahashi

Kazuo Kobayashi

Kazuya Yamagata

Kazuyo Tsushita

Keiichiro Matoba

Keiko Arai

Keisuke Kuwahara

Ken Kato

Ken Sugimoto

Ken Suzuki

Kengo Azushima

Ken‐Ichi Aihara

Kenji Ashida

Kenji Sugawara

Kentaro Doi

Kentaro Watanabe

Keyu Guo

Koichi Gonda

Koichi Kato

Koki Mise

Kong Y. Chen

Kosuke Yamahara

Kouichi Tamura

Kousuke Yamahara

Kuan‐Hung Lin

Kun Gao

Kun Yang

Kwame Yeboah

Kyoichiro Tsuchiya

Kyoung Hwa Ha

Kyu Yong Cho

Kyuho Kim

Lei Wang

Liang Zheng

Liang‐Yu Lin

Liliana Rogozea

Lin Li

Lindawati S. Kusdhany

Ling Wang

Liping Liu

Maciej Tarnowski

Makiko Naka Mieno

Makoto Daimon

Malgorzata Jamka

Marcos Pazos

Marcus Law

Marian Tanofsky Kraff

Mariia Matveeva

Masahide Hamaguchi

Masahiro Yamamoto

Masahito Ogura

Masaji Sakaguchi

Masakatsu Sone

Masaki Igarashi

Masaki Kobayashi

Masanori Emoto

Masao Toyoda

Masaru Akiyama

Masashi Demura

Masashi Shimoda

Masashi Yoshida

Masato Takeuchi

Masatoshi Nomura

Masaya Sakamoto

Masayuki Baba

Masayuki Hosoi

Mashito Sakai

Masoud Mohammadnezhad

Masumi Hara

Mayu Watanabe

Megu Yamaguchi‐Baden

Mehran Rahimlou

Mengyao Zeng

Merve Ayik Türk

Michiaki Fukui

Michihiro Hosojima

Michio Shimabukuro

Mika Yamauchi

Minako Imamura

Mingliang Zhang

Mitsuhiko Noda

Mitsunori Fujino

Mitsuyoshi Takahara

Mohammad Alfhili

Mototsugu Nagao

Muhammad Shahzad Aslam

Munehide Matsuhisa

N. K. Elnaggar

Nagaaki Tanaka

Naiming Zhou

Nan Hee Kim

Naoki Kashihara

Naoko Iwasaki

Naoto Katakami

Naoto Kubota

Naoya Yahagi

Nasim Abedimanesh

Natalia Nowak

Nicholas Carr

Nichole Kelly

Ninon Foussard

Nizamettin Bozbay

Nobuhiro Shojima

Nobuhisa Nakamura

Nobukazu Miyamoto

Norihiro Nishioka

Norikazu Maeda

Noriko Asahara

Noriko Ihana‐Sugiyama

Noriko Kodani

Norio Abiru

Osamu Arisaka

Özge Besci

Paola Algeri

Pau‐Chung Chen

Paul Hsu

Payam Hosseinzadeh Kasani

Pei‐Wen Wang

Ph Wang

Prasert Sakulsriprasert

R. Saucedo

Rajeev Chawla

Rajesh Singh

Rakhi Gaur

Ren Qingwen

Ren‐Hua Chung

Renu Sah

Rian Adi Pamungkas

Rimei Nishimura

Rongyun Wang

Roozbeh Naemi

Ruhi Sikka

Rumi Fujikawa

Runtang Meng

Ryoji Nagai

Ryotaro Bouchi

Saliha Aksun

Sally Mohammed El‐Hefnawy

Sang‐Man Jin

Sarah Gregory

Sathish Thirunavukkarasu

Satoru Tsujii

Satoru Yamada

Satoshi Hirayama

Satoshi Miyamoto

Satoshi Usami

Sen Zhang

Seok Hui Kang

Seung Min Chung

Sh Wang

Shailesh Kumar Samal

Shan Liu

Shaoyuan Chuang

Sharmin Shabnam

Sheyu Li

Shigeru Aoki

Shiho Fujisaka

Shihua Shi

Shimosawa Tatsuo

Shimpei Fujimoto

Shin Takasawa

Shin Tsunekawa

Shin‐Ichi Araki

Shinji Kume

Shinsuke Noso

Shinya Furukawa

Shirley Li

Shiro Tanaka

Sho Tano

Shota Moyama

Shu Meguro

Shubham Kumar

Shuhei Nakanishi

Shun Matsuura

Shunichi Otaka

Shunichiro Asahara

Shun‐Ichiro Asahara

Si Jin

Sodai Kubota

Stefano Passanisi

Stephane Allouche

Sue Lynn Lau

Suheir Ereqat

Suleyman Cemil Oglak

Suma Elumalai

Sumaiyah Mat

Sumito Ogawa

Sunil Deshpande

Suresh Sukumar

Swayam Prakash Srivastava

Swayam Srivastava

Sylvester Lokpo

Tadahiro Kitamura

Tadayoshi Karasawa

Takaaki Murakami

Takahisa Deguchi

Takahisa Hirose

Takao Nammo

Takao Urabe

Takashi Matsuoka

Takashi Miida

Takashi Nomiyama

Takashi Sozu

Takayoshi Sasako

Takayuki Miki

Takehiro Kato

Takehiro Sugiyama

Takeshi Horii

Takeshi Inagaki

Takeshi Iwase

Takeshi Kikuchi

Takeshi Kurose

Takeshi Miyatsuka

Takeshi Onoue

Takuhito Shoji

Takumi Kawaguchi

Takuya Fujimaru

Tang Yi Liao

Tania Romo‐Gonzalez

Tatiana L. Karonova

Tatsuhiko Urakami

Tatsuhito Himeno

Tatsumi Moriya

Tatsuya Kondo

Taura Daisuke

Terisha Ghazi

Teruo Jojima

Tess Chee

Tetsuya Mizoue

Thomas Van Sloten

Ting Wu

Ting‐Wei Lee

Tomoya Mita

Tomoyasu Fukui

Tomoyuki Kawamura

Tong Yue

Toshiaki Ohkuma

Toshihito Fujii

Toshikazu Kondo

Toshinari Takamura

Toshinori Imaizumi

Toshiyasu Sasaoka

Touch Khun

Toyoshi Inoguchi

Tsutomu Hirano

Tuan Van Nguyen

Vasudha Ahuja

Viktor Rotbain Curovic

Vildan Kocatepe

Walter L. Swardfager

Wan‐Wan Lin

Weihua Yang

Wei‐Min Chu

Weiwei Mao

Weiwei Qin

Wenan Xu

Wenrui Shi

Wensheng Lu

Won‐Il Choi

Xianglian Peng

Xiangyu Meng

Xiao Li Shen

Xiaoling Cai

Xiaomu Kong

Xiaoyan Huang

Xinwu Lu

Xiu‐Feng Huang

Xiumei Wu

Xu Zhu

Xue‐Lian Zhang

Yan Yang

Yanbing Li

Yang‐Ching Chen

Yasuaki Hayashino

Yasuharu Ohta

Yasuharu Tabara

Yasuhiko Yamamoto

Yasuko K. Bando

Yasumichi Mori

Yasuo Terauchi

Yasuo Zenimaru

Ye Liu

Yiming Mu

Yi‐Rong Chen

Yixing Yuchi

Yohei Ueda

Yoichi Oikawa

Yong Kyung Kim

Yong Zhang

Yongdi Wang

Yoshiaki Morishita

Yoshifumi Kasuga

Yoshifumi Saisho

Yoshifumi Tamura

Yoshihiro Takamura

Yoshihito Fujita

Yoshikazu Tamori

Yoshiki Kusunoki

Yoshimasa Aso

Yoshinari Obata

Yoshio Fujitani

Yoshio Nagai

Yoshiro Kato

Yoshitaka Hashimoto

Yoshitaka Hayashi

Yoshitsugu Chigusa

Yoshiya Hosokawa

Yosuke Okada

Youqian Zhang

Youyi Wu

Yu Ding

Yu Feng

Yu Liu

Yu Zhang

Yue Chen

Yuji Hataya

Yuji Yamazaki

Yujiro Hayashi

Yujiro Nakano

Yukako Tatsumi

Yukihiro Fujita

Yukiko Okazaki

Yukio Horikawa

Yuko Ishida

Yunyi Le

Yunze Li

Yushi Hirota

Yuta Takagaki

Yuya Nishida

Yuya Tsurutani

Zhangrong Xu

Zhangxia Cui

Zhen Zhao

Zhongyuan Xia

